# Different Desensitization Patterns for Sensory and Vascular TRPV1 Populations in the Rat: Expression, Localization and Functional Consequences

**DOI:** 10.1371/journal.pone.0078184

**Published:** 2013-11-08

**Authors:** Ágnes Czikora, Ibolya Rutkai, Enikő T. Pásztor, Andrea Szalai, Róbert Pórszász, Judit Boczán, István Édes, Zoltán Papp, Attila Tóth

**Affiliations:** 1 Division of Clinical Physiology, Institute of Cardiology, University of Debrecen, Debrecen, Hungary; 2 Department of Pharmacology and Pharmacotherapy, Institute of Pharmacology, University of Debrecen, Debrecen, Hungary; 3 Deparment of Neurology, University of Debrecen, Debrecen, Hungary; 4 Research Centre for Molecular Medicine, Medical and Health Science Center, University of Debrecen, Debrecen, Hungary; Indiana University School of Medicine, United States of America

## Abstract

**Background and purpose:**

TRPV1 is expressed in sensory neurons and vascular smooth muscle cells, contributing to both pain perception and tissue blood distribution. Local desensitization of TRPV1 in sensory neurons by prolonged, high dose stimulation is re-engaged in clinical practice to achieve analgesia, but the effects of such treatments on the vascular TRPV1 are not known.

**Experimental approach:**

Newborn rats were injected with capsaicin for five days. Sensory activation was measured by eye wiping tests and plasma extravasation. Isolated, pressurized skeletal muscle arterioles were used to characterize TRPV1 mediated vascular responses, while expression of TRPV1 was detected by immunohistochemistry.

**Key results:**

Capsaicin evoked sensory responses, such as eye wiping (3.6±2.5 versus 15.5±1.4 wipes, p<0.01) or plasma extravasation (evans blue accumulation 10±3 versus 33±7 µg/g, p<0.05) were reduced in desensitized rats. In accordance, the number of TRPV1 positive sensory neurons in the dorsal root ganglia was also decreased. However, TRPV1 expression in smooth muscle cells was not affected by the treatment. There were no differences in the diameter (192±27 versus 194±8 µm), endothelium mediated dilations (evoked by acetylcholine), norepinephrine mediated constrictions, myogenic response and in the capsaicin evoked constrictions of arterioles isolated from skeletal muscle.

**Conclusion and implications:**

Systemic capsaicin treatment of juvenile rats evokes anatomical and functional disappearance of the TRPV1-expressing neuronal cells but does not affect the TRPV1-expressing cells of the arterioles, implicating different effects of TRPV1 stimulation on the viability of these cell types.

## Introduction

The therapeutic potential of TRPV1 modulation in various painful states has been recognized in a series of publications some 40 years ago [1]–[4]. In these early works capsaicin specific responses were related to a specific receptor, which could be desensitized by the application of high dose of capsaicin at an early phase of the life of the rat. These robust changes in sensory function led to the recognition of pharmacological approach to manipulate nociceptors [5]. Capsaicin desensitization therefore become a powerful approach to characterize peripheral nociceptors and to achieve topical analgesia. Although molecular identification of the receptor responsible for capsaicin mediated responses [6] resulted in a rise in therapeutic interest in systemic TRPV1 modulation to inhibit the pain pathway, no successful antagonists were introduced to the market [7], probably due to the unfavorable side effects of the systemic treatment [8]. On the other hand, local capsaicin desensitization recently re-emerged as a therapeutic method to selectively defunctionalize capsaicin expressing sensory neurons and to achieve local analgesia [9], [10].

TRPV1 regulates cellular Ca^2+^ levels via direct permeation (P_Ca_/P_Na_∼10) [6], which concomitantly down-regulates its own activity. Among the Ca^2+-^activated enzymes that are believed to play pivotal roles in this TRPV1 acute desensitization process is the Ca^2+^/calmodulin-dependent Ser/Thr phosphatase 2B, calcineurin [11]–[13], which dephosphorylates TRPV1 receptors. Conversely, phosphorylations at several consensus sites for protein kinase C (PKC) [14]–[16] and cAMP-dependent protein kinase A (PKA) [17], [18] can reduce the Ca^2+^- mediated desensitization of TRPV1. Thus, the dynamic balance between the Ca^2+^-dependent phosphorylation and dephosphorylation of the TRPV1 protein appears to play a critical role in the acute desensitization of TRPV1 [12].

In contrast with the acute desensitization, neonatal capsaicin treatment results in the deletion of TRPV1 expressing peripheral sensory neurons [19], [20]. Capsaicin evoked sensory neuronal cell death may be mediated by Ca^2+^ overload of the TRPV1 expressing cells [21].

Activation of TRPV1 leads to central (pain) and to local “sensory-efferent” effects [22]. These include the release of vasoactive agents (such as calcitonin gene-related peptide) from sensory neurons and subsequent vasorelaxation [23]. In recent years, TRPV1 expression has been identified in many cells types in addition to sensory neurons as referenced by Keeble et al in recent reviews [24], [25]. In particular, TRPV1 expression was detected in various cell types in the brain [26], and in arteriolar smooth muscle [27]. In accordance with this latter non-neuronal localization, activation of TRPV1 resulted in a substantial vasoconstriction *in vivo* and *in vitro* [27], [28] through direct activation of endogenous TRPV1 in vascular smooth muscle cells [29].

The effects of capsaicin desensitization were investigated here. Capsaicin desensitization is the only clinically available, approved and effective treatment option to regulate TRPV1 mediated sensory functions. Since functional TRPV1 expression has been identified in non-neuronal cells, these receptors may have physiological functions which may also be modulated by therapeutic capsaicin applications. Morphological and functional studies were performed to reveal differences between desensitization of sensory neurons and vascular smooth muscle cells. Our data suggest that capsaicin desensitization has only a transient effect on the vascular TRPV1. Therefore, vascular TRPV1 function is maintained even if the same treatment depletes sensory neurons.

## Methods

### Animals, anaesthesia and general preparation in the *in vivo* experiments

Male Wistar Kyoto (WKY/NCrl) rats obtained from Charles River (Isaszeg, Hungary) were fed by CRLT/N chow (Szinbad Kft, Godollo, Hungary). Experiments were performed on male Wistar rats weighing about 250 g at the beginning of the experiments raised on a standard laboratory food and water *ad libitum*. Anaesthesia was performed with 50 mg/kg i.p. thiopental. Animal experiments were carried out and approved by the Hungarian Ministry of Rural Development and by the University of Debrecen, Medical and Health Science Center (Registration number: 31/2007/DE MÁB), and were in accordance with the standards established by the National Institutes of Health.

### Capsaicin pretreatment of rats

Newborn rats (at day 14 of life) were pretreated with Diaphyllin (Richter, Hungary), Bricanyl (Astra Zeneca, Hungary), and atropine (Egis, Hungary, 100 g/0,1 ml i.p.). Ten minutes later animals were injected with capsaicin (subcutaneously). The procedure was repeated for a total of five consecutive days. The total dose of capsaicin was 300 mg/kg, administered on a dose schedule of 10 mg/kg, 20 mg/kg; 50 mg/kg; 100 mg/kg; and 120 mg/kg on days 1 through 5, respectively. Rats were then kept in the animal facility for 10 weeks when experiments were performed. The weight of the rats was in the range of 360–492 g at this time.

### Measurement of capsaicin evoked sensory irritation

One drop (10 µl) of capsaicin solution (50 µg/ml in physiological saline) was put into the right or left conjunctiva of the rat, in a random order. The number of eye wipes was counted during 60 s. Rats were scarified after capsaicin treatments.

### Measurement of capsaicin evoked involuntary sensory neuronal functions

Plasma extravasation was measured according to Pinter et al. [30]. Rats were anesthetised and a tail vein was cannulated for the injection of Evans blue dye (30 mg/kg). One min after Evans blue administration capsaicin was injected (1 mg/kg, i.v.). Rats was sacrificed by transcardiac perfusion with 50 ml of 0.9% w/v saline, at 37°C, through the left cardiac ventricle 10 min after injection of Evans blue. The urinary bladder was removed and weighed, and the Evans blue was extracted in 1 ml of formamide for 24 h. Evans blue content was determined by spectrophotometry (at 620 nm). Plasma extravasation was expressed as the content of Evans blue dye in micrograms per gram of wet tissue.

### Preparation of cannulated skeletal muscle arterioles

Isolation of the skeletal (gracilis) muscle arterioles of the rat and diameter measurement of the arterioles were performed as described earlier [12]. The internal diameters of the cannulated skeletal muscle (m. gracilis) arterioles were determined at the midpoint of the arteriolar segment by videomicroscopy. Cannulated arterioles were incubated in a physiological solution (PSS, composition in mM: 110 NaCl, 5.0 KCl, 2.5 CaCl_2_, 1.0 MgSO_4_, 1.0 KH_2_PO_4_, 5.0 glucose and 24.0 NaHCO_3_ equilibrated with a gas mixture of 10% O_2_ and 5% CO_2_, 85% N_2_, at pH 7.4.). Experiments were started after the development of a spontaneous tone in response to intraluminal pressure of 80 mmHg. First, acetylcholine (1 nM–10 µM) was used to determine dilatative capacity (acetylcholine causes endothelium dependent vasodilatation), and then norepinephrine (1 nM–10 µM) was applied to measure maximal constrictive response (norepinephrine causes smooth muscle dependent constriction). Vascular autoregulation (myogenic response) were also determined. Intraluminal pressure was increased from 20 to 120 mmHg in 20 mmHg increments and arteriolar diameter was measured after 4 min incubations at each intraluminal pressures. These measurements were performed in PSS to assess the active myogenic response. Passive diameter was determined in Ca^2+^ free PSS at the end of the experiments. Changes in diameter in response to a TRPV1 agonist was tested with cumulative dose (capsaicin, 1 nM–30 µM).

### Immunohistochemical procedures

Tissue sections were prepared as detailed earlier [27]. In short, rat skeletal muscle (m. gracilis), dorsal root ganglions (thoracic) were dissected from Wistar rats and were embedded in Tissue-Tek O.C.T compound (Electron Microscopy Sciences, Hatfield, PA, USA). Cryostat sections (thickness 10 µm) were placed on adhesive slides and fixed in acetone for 10 min. The slices were blocked with normal goat sera (1.5% in PBS, Sigma, St. Louis, MO, USA) for 20 min and stained with an anti-capsaicin receptor antibody (PC 547 (rabbit), Calbiochem, San Diego, CA) at a 1∶500 (for gracilis muscle) and 1∶200 dilution (for dorsal root ganglia), and co-stained with smooth muscle actin antibody (NCL-SMA, dilution, 1∶50; Novocastra Laboratories, New Castle, UK) or with a neurofilament-specific antibody (dilution, 1∶50; Sigma) in the blocking buffer. Then, the slices were incubated with biotinylated anti-rabbit (Jackson, Suffolk, England, 1∶200) and FITC conjugated anti-mouse antibodies (Jackson, Suffolk, England, 1∶100). Biotinylated antiobody was detected by Cy3 conjugated streptavidine (Jackson, Suffolk, England, 1∶500). The pictures were captured by a Scion Corporation (Frederick, MA) digital camera attached to a Nikon Eclipse 80i fluorescent microscope (Nikon, Tokyo, Japan) further processed by ImageJ (freeware from www.nih.gov) software.

### Materials and solutions

Chemicals were from Sigma-Aldrich (St. Louis, MO, USA). Capsaicin (8-Methyl-N-vanillyl-*trans*-6-nonenamide) was dissolved in ethanol. Norepinephrine and acetylcholine were dissolved in distilled water. Evans Blue concentration was measured by using a NOVOstar plate reader.

### Data analysis and statistical procedures

Arteriolar diameter was determined by measuring the distances between the intraluminal sides of the arteriolar wall (inner diameter). Data are shown as average diameter ± S.E.M. Statistical analysis was made by Microsoft Excel using Student's t-tests. P-values<0.05 were considered to be significant.

## Results

### Effects of desensitization on capsaicin-evoked eye-wiping movements

**Figure 1:**
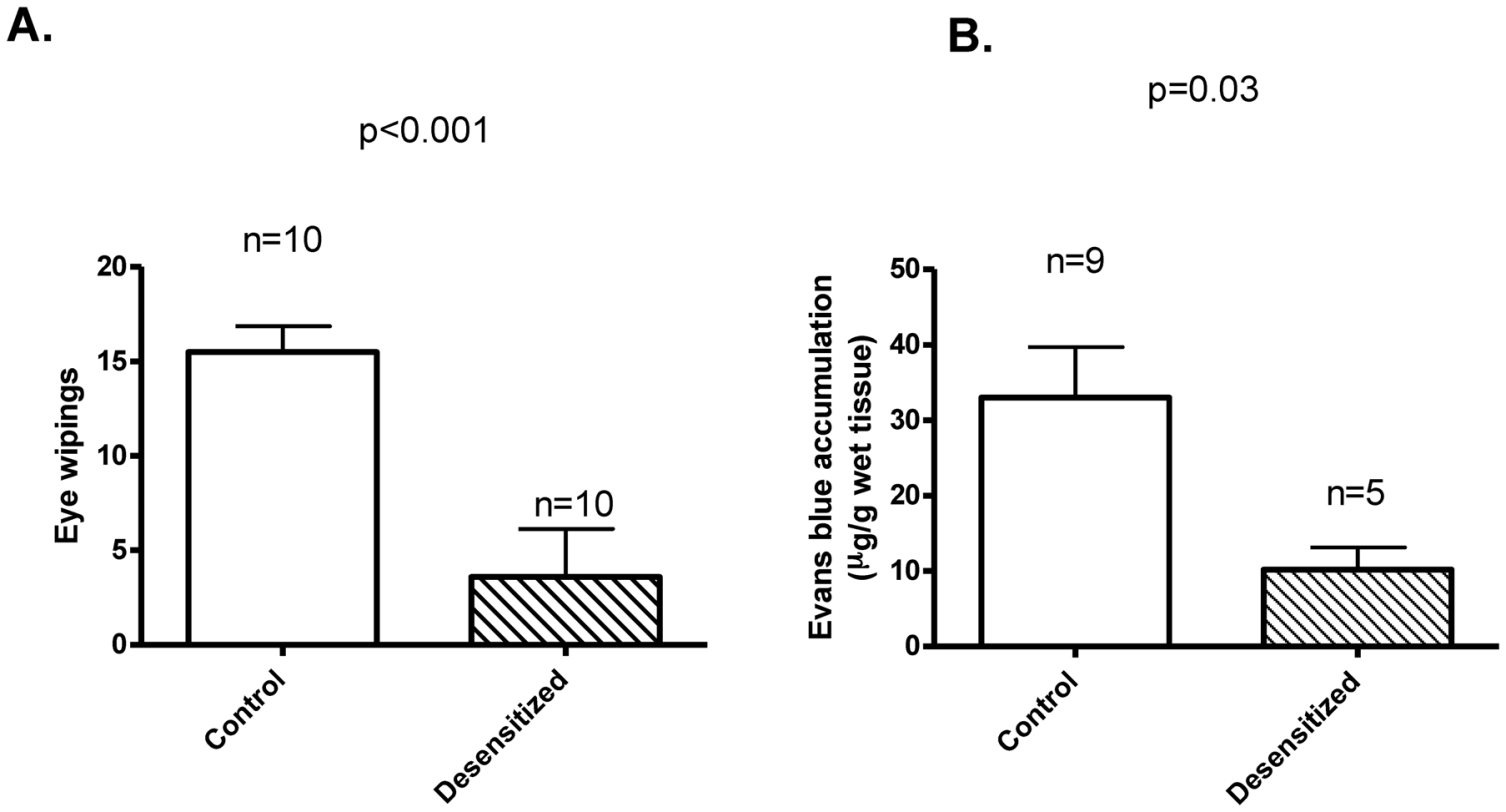
Effects of neonatal capsaicin desensitization on sensory functions. Rats were treated with saline (Control) or with capsaicin (Desensitized) at 14 days of life. Sensory functions were measured 10 weeks after capsaicin treatment. (A) Eye wiping (a measure of capsaicin evoked sensory irritation) was reduced in desensitized rats (n = 10 in both groups, p<0.001). (B) Plasma extravasation was similarly reduced (Evans blue accumulation, a measure of capsaicin mediated neurogenic inflammation) (n = 9 for Control and n = 5 for Desensitized, p = 0.03).

Sensory irritation was assessed by eye wiping assays. Capsaicin evoked a high number of wiping movements in control rats (15.5±1.4 wiping movements, n = 10), while desensitized rats (neonatal capsaicin application) were almost insensitive to the same treatment (3.6±2.5 wipes, n = 10; p<0,001, Fig. 1A).

### Effects of desensitization on capsaicin – induced plasma extravasation

Systemic activation of TRPV1 expressing sensory neurons by capsaicin resulted in an increase in Evans blue accumulation in the urinary bladder of control rats (33±7 µg/g wet tissue; n = 9, Fig. 1B). Neonatal capsaicin desensitization significantly attenuated this response (Evans blue accumulation decreased to 10±3 µg/g wet tissue; n = 5, p = 0,03 versus control, Fig. 1B).

### Immunochemistry of TRPV1

**Figure 2:**
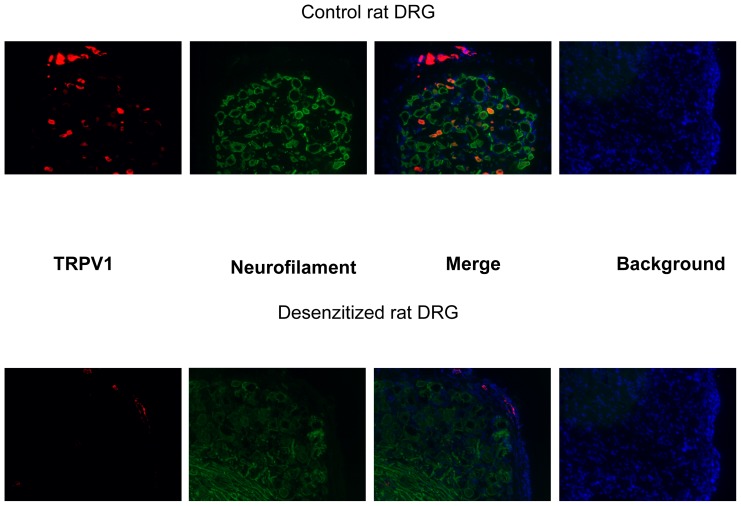
Effects of neonatal capsaicin desensitization on sensory TRPV1 expression. TRPV1 expression was examined in the dorsal root ganglia of control and capsaicin desensitized (neonatal capsaicin treatment) adult rats. Tissue sections were probed with antibodies specific to TRPV1 (red) and neurofilament (green). TRPV1 expression was present in a subset of sensory neurons in control rats, which was missing in desensitized rats within the dorsal root ganglia. However, TRPV1 immunoreactivity was also found in non-neuronal cell types, located at the outer regions of ganglia (regions occupied by sensory neurons are indicated by the freehand drawing). Images are representative of at least 10 sections.

**Figure 3:**
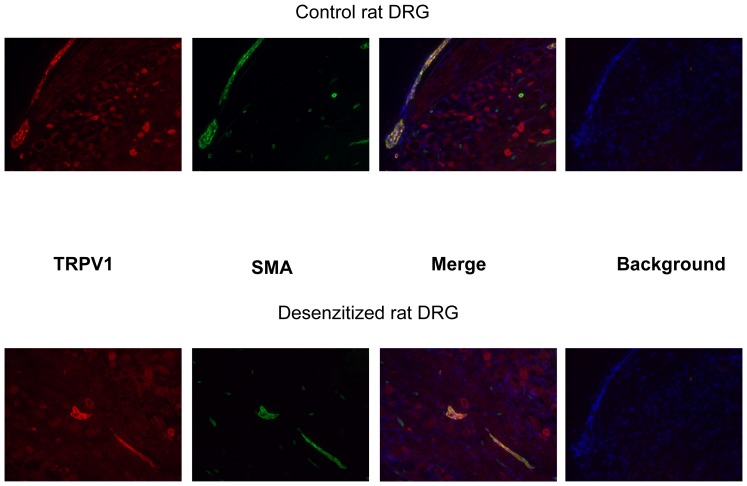
TRPV1 immunoreactivity in vascular smooth muscle cells. Rat dorsal root ganglia sections were co-stained by anti TRPV1 (red) and anti vascular smooth muscle actin (SMA, green) antibodies. Some of the TRPV1 positive cells were co-stained by the antibody specific to vascular smooth muscle actin, suggesting TRPV1 expression in this cell type. Moreover, vascular TRPV1 expression was not affected by neonatal capsaicin treatment (note similar TRPV1 staining in SMA positive regions of control and desensitized rats). Images are representative of at least 6 sections.

**Figure 4:**
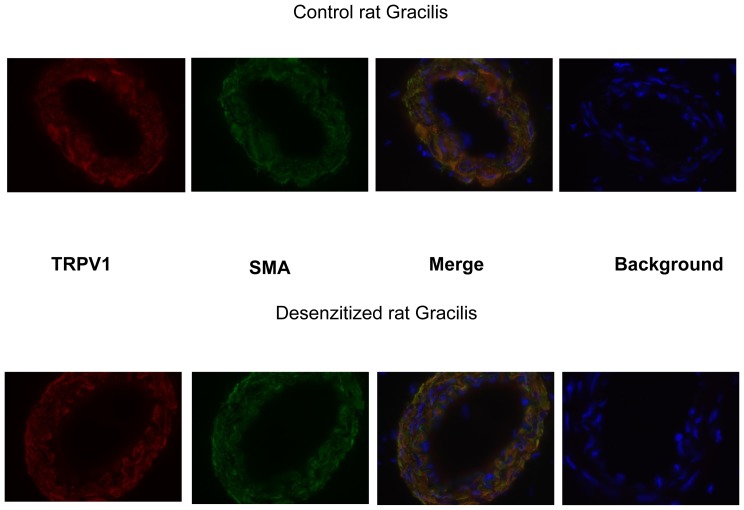
Effects of neonatal capsaicin desensitization on vascular TRPV1 expression. Skeletal muscle (gracilis muscle of the rat) tissue sections were stained with anti-TRPV1 (red) and anti-vascular smooth muscle actin (SMA, green) antibodies. There appeared a complete overlap of the staining patterns (merged images). Arteriolar TRPV1 expression was not affected by neonatal capsaicin desensitization, similarly to the vascular TRPV1 in the dorsal root ganglia. Images are representative of at least 10 sections.

Dorsal root ganglia (DRG) were isolated from control and desensitized rats and TRPV1 expression was evaluated by immunohistochemistry. TRPV1 expression was found in a subset of neurons (neurons were visualized by neurofilament specific staining) in control rats but was missing in desensitized rats (Fig. 2). However, TRPV1 like immunostaining was also found in blood vessels associated with the DRGs, irrespective to the desensitization procedure. The localization of that TRPV1 expression to vascular smooth muscle cells was tested by the co-application of antisera against TRPV1 and vascular smooth muscle actin (Fig. 3). Indeed, some of the TRPV1 immunoreactivity overlapped with vascular smooth muscle specific staining. We thus find that, that while neonatal capsaicin treatment was found to be effective to eliminate TRPV1 immunoreactivity in sensory neurons, it was without effects on the vascular smooth muscle cell located TRPV1 immunoreactivity (Fig. 3). Similarly, TRPV1 expression was not affected by neonatal capsaicin treatment in the gracilis muscle of the rat (Fig. 4).

### Physiological effects of neonatal capsaicin desensitization on isolated skeletal muscle arterioles

**Figure 5:**
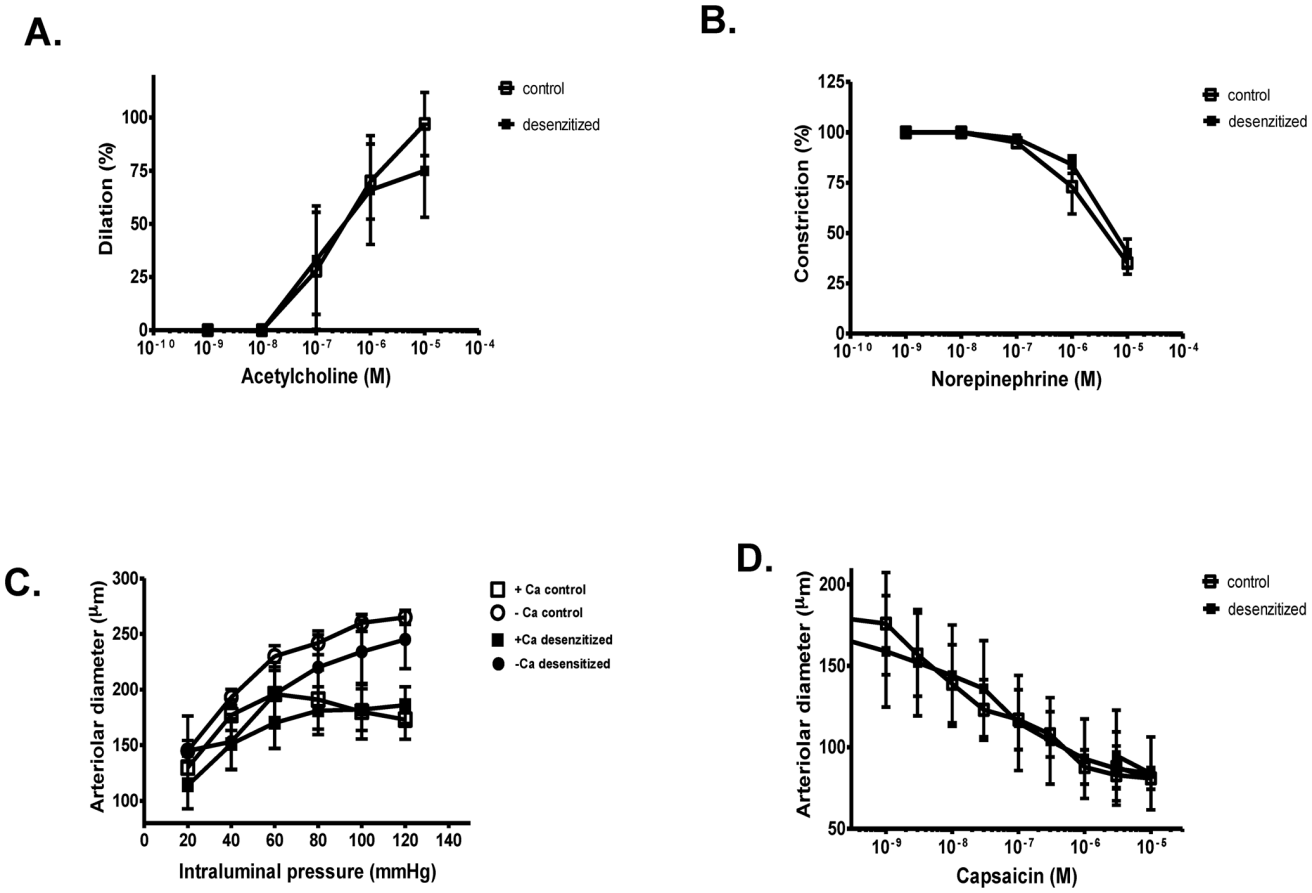
Functional effects of neonatal capsaicin desensitization on arteriolar TRPV1. Effects of neonatal capsaicin desensitization were characterized on the functional properties of isolated, cannulated skeletal muscle arterioles. Endothelial responses were tested by the application of acetylcholine (Panel A, values normalized to the maximal acetylcholine mediated dilations, symbols represent the mean ± SEM, n = 4 for the control and n = 4 for the desensitized). Smooth muscle functions were assessed by norepinephrine (Panel B, symbols represent the mean ± SEM of n = 4 determinations in both cases). The spontaneous myogenic tone (Panel C, symbols represent the mean ± SEM of n = 4 determinations for all groups) was tested by the contractile response developing in response to increasing intraluminal pressure (from 20 mmHg to 120 mmHg in 20 mmHg increments). Finally, capsaicin mediated responses were also tested by the cumulative application of the drug. Capsaicin evoked a dose-dependent constriction, supporting a physiological role for this receptor in vascular smooth muscle cells, which was not affected by the desensitization protocol (Panel D, symbols represent the mean ± SEM of n = 4 determinations for both groups).

There were no significant differences between control and desensitized rats in acetylcholine mediated dilation (Fig. 5A), norepinephrine mediated constriction (Fig. 5B) and vascular autoregulation (spontaneous myogenic response, Fig. 5C) in isolated skeletal muscle resistance arteries. Moreover, capsaicin mediated arteriolar constrictions were also unaffected in both groups of rats (Fig. 5D), which was in contrast with the capsaicin mediated sensory functions (Fig. 1).

**Figure 6:**
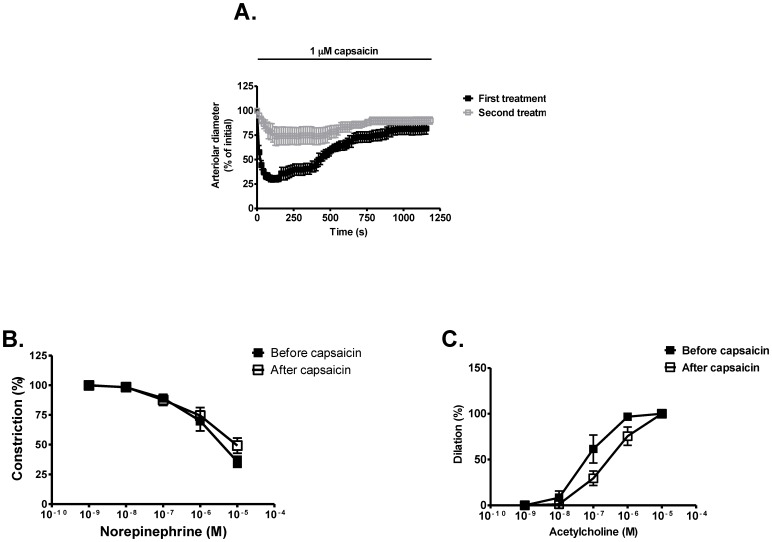
Functional effects of acute capsaicin desensitization on vascular TRPV1. The short-term effects of capsaicin were tested here. Arterioles were isolated from control rats and were treated with capsaicin (1 µM, First treatment). Capsaicin was washed away after the 20 min long treatment and the arterioles were incubated in the physiological buffer alone for 40 min (regeneration). Then capsaicin effects we re-tested (Second treatment) to estimate the level of tachyphylaxis (n = 6 arteries were tested, symbols represent the mean ± SEM of time-matched arteriolar diameter). Acute desensitization to capsaicin was apparent upon the first treatment (decrease and then increase in arteriolar diameter in the continuous presence of capsaicin, maximal decrease was 70±3% at 95 s after capsaicin application, Panel A). The magnitude of the response was decreased upon the second capsaicin treatment on the same arterioles (tachyphylaxis, decreased response to repeated capsaicin stimuli, maximal decrease was 27±9% at 125 s after capsaicin application, Panel A). This acute capsaicin desensitization (20 min treatment) did not affect norepinephrine mediated constrictions (symbols represent the mean ± SEM of n = 6 determinations, Panel B) or acetylcholine mediated dilations (symbols represent the mean ± SEM, n = 6, Panel C).

Direct application of capsaicin (1 µmol/L) resulted in a transient constriction (maximal decrease of arteriolar diameter was 70±3% at 105 s) followed by a dilation in the continuous presence of capsaicin (constriction at the end of the 20 min long incubation was only 8±6%) (Fig. 6A). Besides to the acute desensitization (desensitization in the presence of the agonist) tachyphylaxis (desensitization to the repeated application of the agonist) was also determined. Although tachyphylaxis was apparent upon the second capsaicin challenge (first versus second treatment on Fig. 6A), capsaicin treatment did not eliminate capsaicin responsiveness completely (maximal constriction upon second capsaicin treatment was 26±9% at 270 s, while constriction was only 10±4% at the end of the 20 min long treatment, Fig. 6A). To test if capsaicin mediated desensitization may affect the viability of the different vascular cell types, smooth muscle (norepinephrine, Fig. 6B) and endothelium (acetylcholine, Fig. 6C) mediated responses were also tested. Norepinephrine mediated responses were almost identical (Fig. 6B), while the limited shift in acetylcholine responses (Fig. 6C) did not reach the level of significance (p>0.05), suggesting that acute *in vitro* TRPV1 desensitization did not have a significant effect of TRPV1 independent functions of skeletal muscle arterioles.

## Discussion

Capsaicin containing herbs were used from the prehistoric era in the medicinal efforts to relieve pain and inflammation [31]. Capsaicin treatment was introduced to eliminate sensory responses about fifty years ago [3] and these experiments lead to the identification of primary nociceptors [5]. Cloning of the receptor (named as TRPV1) and the development of knock out models confirmed its role in inflammatory hyperalgesia [32]. It was even proposed that there are endogenous ligands (endovanilloids) binding to the capsaicin binding site of TRPV1 [23], although the physiological relevance of these proposed endovanilloids to modulate TRPV1 are still under debate, especially in skeletal muscle arterioles [33].

TRPV1 appeared to be a sensory neuronal specific receptor, which has a role in nociception and can be selectively targeted by small molecules. Not surprisingly, TRPV1 become a pharmaceutical target to develop a new generation of painkillers as well as to treat other therapeutic conditions [7]. Although almost all of the major pharmaceutical companies initiated a TRPV1 research program, these efforts have not yet produced approved drugs [34].

The apparent failure of introduction of TRPV1 antagonists into the clinical practice is probably not the result of insufficient potency of the developed antagonists [34]. Nonetheless, clinical application of the TRPV1 agonists capsaicin and resiniferatoxin offer an alternative to TRPV1 antagonists for treating challenging painful conditions such as neuropathic pain [10], [35]. It is important to note that therapy to achieve desensitization is different from that based on the application of antagonists in terms of the potential side effects. Antagonists may cause side effects if their receptors play a significant physiological role, while desensitizing agonists may activate receptors which are expressed, but without much physiological role. Interestingly, while recent data suggested that TRPV1 may be expressed in other cell types than sensory neurons (see Keeble et al [24], [25] for references and [36]), the potential side effects of capsaicin desensitization had not been tested on these receptors.

Selective elimination of TRPV1 positive sensory neurons and attenuation of capsaicin evoked sensory neuron mediated effects were found following systemic neonatal capsaicin treatment confirming earlier data [37], [38]. Effects of this treatment on the vascular smooth muscle located TRPV1 was addressed here for the first time. It was found that neonatal capsaicin treatment does not affect arteriolar TRPV1 function and expression. It is in accordance with the limited if any permanent vascular effects of neonatal capsaicin desensitization.

It has been suggested that capsaicin has biphasic effects on the vasculature: at lower concentrations, capsaicin (up to 10 nM) evokes vasodilation in skin due to sensory nerve activation, while higher concentrations (0.1–1 µM) lead to substantial constrictions in skeletal muscle arterioles due to non-neuronal TRPV1 stimulation [27]. This apparent difference in sensitivity may be due to (i) receptor sensitivity or (ii) a difference in TRPV1 receptor density in skin and skeletal muscle arterioles as been concluded by Fernandes et al [25]. As the matter of the first hypothesis a recent study designed to detect pharmacological differences between TRPV1 populations in the sensory nerves and in the arteriolar smooth muscle proved that these TRPV1 populations are pharmacologically different: some TRPV1 agonists were irritative (a measure of sensory neuronal TRPV1 activation), but without vasoconstrictive effects [29]. Capsaicin evoked vasoconstriction was mediated by direct activation of arteriolar smooth muscle located TRPV1 and was missing in TRPV1 knock out mice, suggesting that both the irritation (sensory neuronal response) and vasoconstriction (smooth muscle response) are mediated by TRPV1 [29]. As the matter of the second hypothesis (different expression of TRPV1 in perivascular nerves) a clear tissue specific difference was described. Arteries in the skin were densely innervated by TRPV1 positive fibers, in contrast with arteries in the skeletal muscle [27]. This fact suggests that sensory neuron mediated dilation plays a dominant role in the skin upon TRPV1 stimulation, but may have a limited effect in the skeletal muscle.

TRPV1 was found to be important in inflammation affecting arteriolar diameter. The most prominent contribution of TRPV1 stimulation to the neurogenic inflammation is the local release of neuropeptides (such as CGRP and substance P) from the sensory neurons [23], which is missing in capsaicin desensitized rats [5]. However, it was found recently, that TRPV1 stimulation also increases the plasma concentration of PACAP-38, an anti-inflammatory peptide [39], and somatostatin [40] suggesting a complex regulation of neurogenic inflammatory response, as summarized by Alawi and Keeble in an exemplary review [24]. These observations suggest that TRPV1 has a role in vascular inflammation. Chronic physiological activation of TRPV1 expressing sensory neurons may contribute to the inflammatory response and to painful effects such as hyperalgesia and allodynia [24]. In particular, diabetic rats showed increased TRPV1 function (while TRPV1 expression decreased) in the dorsal root ganglia [41], a feature which is similarly present in human diabetic patients [42]. This represents a local feedback regulating TRPV1 activity: TRPV1 activation causes a desensitization of the TRPV1 itself (limiting hyperalgesia and allodynia), which is mediated by decreased expression of TRPV1 in the tissues. This suggests that TRPV1 desensitization has an effect on tissue perfusion. In particular, diabetic or other kind of neuropathies result in a decrease in TRPV1 expressing sensory neurons, mediating vasodilation. As a result, patients with diabetes may have an impaired regulation of blood flow leading to the dominance of smooth muscle TRPV1 mediated vasoconstrictions over the physiological sensory neuronal TRPV1 mediated vasodilations. This altered vascular response may contribute to the progression of the disease in the skin.

The apparent difference between sensory neurons and vascular smooth muscle cells in response to high capsaicin stimulation (desensitization) described in this work may be explained by at least two mechanisms. First, vascular smooth muscle cells are able to proliferate, therefore the seriously damaged TRPV1 expressing cells may be replaced. Alternatively, capsaicin damage is less severe in vascular smooth muscle cells compared to sensory neurons. To test this latter possibility, isolated arteries were treated with capsaicin *in vitro*. Arteriolar TRPV1 was rapidly (within minutes) desensitized to capsaicin and capsaicin treatment did not affect TRPV1 independent functions, suggesting that capsaicin mediated Ca^2+^ toxicity did not occur in these cells.

We propose that vascular smooth muscle cells can be activated by the application of high doses of capsaicin, but the Ca^2+^ toxicity is limited by fast desensitization, in accordance with previous findings [12]. Vascular smooth muscle cells are therefore protected from prolonged capsaicin treatments and maintain their viability. These cells then are able to regenerate and regain capsaicin responsiveness (and probably their physiological role) within a short period of time. In contrast, sensory neurons seem to be eliminated and not replaced upon neonatal capsaicin treatment. Taken together, our data suggest that capsaicin desensitization is an effective method to specifically regulate sensory functions, in contrast with TRPV1 antagonists, which may affect vascular TRPV1 mediated processes in addition to sensory functions.

## References

[1] Jancso-Gabor A, Szolcsanyi J, Jancso N (1970) Stimulation and desensitization of the hypothalamic heat-sensitive structures by capsaicin in rats. J Physiol 208: 449–459. 5500735 10.1113/jphysiol.1970.sp009130 PMC1348759

[2] Jancso-Gabor A, Szolcsanyi J, Jancso N (1970) Irreversible impairment of thermoregulation induced by capsaicin and similar pungent substances in rats and guinea-pigs. J Physiol 206: 495–507. 5498502 10.1113/jphysiol.1970.sp009027 PMC1348662

[3] Jancso N, Jancso-Gabor A, Szolcsanyi J (1967) Direct evidence for neurogenic inflammation and its prevention by denervation and by pretreatment with capsaicin. Br J Pharmacol Chemother 31: 138–151. 6055248 10.1111/j.1476-5381.1967.tb01984.x PMC1557289

[4] Jancso N, Jancso-Gabor A, Szolcsanyi J (1968) The role of sensory nerve endings in neurogenic inflammation induced in human skin and in the eye and paw of the rat. Br J Pharmacol Chemother 33: 32–41. 4298238 10.1111/j.1476-5381.1968.tb00471.x PMC1570273

[5] Szolcsanyi J (1977) A pharmacological approach to elucidation of the role of different nerve fibres and receptor endings in mediation of pain. J Physiol (Paris) 73: 251–259. 926026

[6] Caterina MJ, Schumacher MA, Tominaga M, Rosen TA, Levine JD, et al (1997) The capsaicin receptor: a heat-activated ion channel in the pain pathway. Nature 389: 816–824. 9349813 10.1038/39807

[7] Wong GY, Gavva NR (2009) Therapeutic potential of vanilloid receptor TRPV1 agonists and antagonists as analgesics: Recent advances and setbacks. Brain Res Rev 60: 267–277. 19150372 10.1016/j.brainresrev.2008.12.006

[8] Gavva NR, Treanor JJ, Garami A, Fang L, Surapaneni S, et al (2008) Pharmacological blockade of the vanilloid receptor TRPV1 elicits marked hyperthermia in humans. Pain 136: 202–210. 18337008 10.1016/j.pain.2008.01.024

[9] Backonja M, Wallace MS, Blonsky ER, Cutler BJ, Malan P Jr, et al (2008) NGX-4010, a high-concentration capsaicin patch, for the treatment of postherpetic neuralgia: a randomised, double-blind study. Lancet Neurol 7: 1106–1112. 18977178 10.1016/S1474-4422(08)70228-X

[10] Noto C, Pappagallo M, Szallasi A (2009) NGX-4010, a high-concentration capsaicin dermal patch for lasting relief of peripheral neuropathic pain. Curr Opin Investig Drugs 10: 702–710. 19579176

[11] Docherty RJ, Yeats JC, Bevan S, Boddeke HW (1996) Inhibition of calcineurin inhibits the desensitization of capsaicin-evoked currents in cultured dorsal root ganglion neurones from adult rats. Pflugers Archiv European journal of physiology 431: 828–837. 8927498 10.1007/s004240050074

[12] Lizanecz E, Bagi Z, Pásztor ET, Papp Z, Edes I, et al (2006) Phosphorylation-dependent desensitization by anandamide of vanilloid receptor-1 (TRPV1) function in rat skeletal muscle arterioles and in Chinese hamster ovary cells expressing TRPV1. Molecular pharmacology 69: 1015–1023. 16338989 10.1124/mol.105.015644

[13] Mohapatra DP, Nau C (2005) Regulation of Ca2+-dependent desensitization in the vanilloid receptor TRPV1 by calcineurin and cAMP-dependent protein kinase. The Journal of Biological Chemistry 280: 13424–13432. 15691846 10.1074/jbc.M410917200

[14] Bhave G, Hu HJ, Glauner KS, Zhu W, Wang H, et al (2003) Protein kinase C phosphorylation sensitizes but does not activate the capsaicin receptor transient receptor potential vanilloid 1 (TRPV1). Proc Natl Acad Sci U S A 100: 12480–12485. 14523239 10.1073/pnas.2032100100 PMC218783

[15] Mandadi S, Numazaki M, Tominaga M, Bhat MB, Armati PJ, et al (2004) Activation of protein kinase C reverses capsaicin-induced calcium-dependent desensitization of TRPV1 ion channels. Cell Calcium 35: 471–478. 15003856 10.1016/j.ceca.2003.11.003

[16] Numazaki M, Tominaga T, Toyooka H, Tominaga M (2002) Direct phosphorylation of capsaicin receptor VR1 by protein kinase Cepsilon and identification of two target serine residues. J Biol Chem 277: 13375–13378. 11884385 10.1074/jbc.C200104200

[17] Bhave G, Zhu W, Wang H, Brasier DJ, Oxford GS, et al (2002) cAMP-dependent protein kinase regulates desensitization of the capsaicin receptor (VR1) by direct phosphorylation. Neuron 35: 721–731. 12194871 10.1016/s0896-6273(02)00802-4

[18] Mohapatra DP, Nau C (2003) Desensitization of capsaicin-activated currents in the vanilloid receptor TRPV1 is decreased by the cyclic AMP-dependent protein kinase pathway. The Journal of Biological Chemistry 278: 50080–50090. 14506258 10.1074/jbc.M306619200

[19] Ralevic V, Karoon P, Burnstock G (1995) Long-term sensory denervation by neonatal capsaicin treatment augments sympathetic neurotransmission in rat mesenteric arteries by increasing levels of norepinephrine and selectively enhancing postjunctional actions. J Pharmacol Exp Ther 274: 64–71. 7616449

[20] Scadding JW (1980) The permanent anatomical effects of neonatal capsaicin on somatosensory nerves. J Anat 131: 471–482. 7216914 PMC1233246

[21] Cortright DN, Szallasi A (2004) Biochemical pharmacology of the vanilloid receptor TRPV1. An update. Eur J Biochem 271: 1814–1819. 15128291 10.1111/j.1432-1033.2004.04082.x

[22] Szolcsanyi J (1983) Tetrodotoxin-resistant non-cholinergic neurogenic contraction evoked by capsaicinoids and piperine on the guinea-pig trachea. Neurosci Lett 42: 83–88. 6657150 10.1016/0304-3940(83)90426-3

[23] Zygmunt PM, Petersson J, Andersson DA, Chuang H, Sørgård M, et al (1999) Vanilloid receptors on sensory nerves mediate the vasodilator action of anandamide. Nature 400: 452–457. 10440374 10.1038/22761

[24] Alawi K, Keeble J (2010) The paradoxical role of the transient receptor potential vanilloid 1 receptor in inflammation. Pharmacology & Therapeutics 125: 181–195. 19896501 10.1016/j.pharmthera.2009.10.005

[25] Fernandes ES, Fernandes MA, Keeble JE (2012) The functions of TRPA1 and TRPV1: moving away from sensory nerves. British Journal of Pharmacology 166: 510–521. 22233379 10.1111/j.1476-5381.2012.01851.x PMC3417484

[26] Toth A, Boczan J, Kedei N, Lizanecz E, Bagi Z, et al (2005) Expression and distribution of vanilloid receptor 1 (TRPV1) in the adult rat brain. Molecular Brain Research 135: 162–168. 15857679 10.1016/j.molbrainres.2004.12.003

[27] Kark T, Bagi Z, Lizanecz E, Pásztor ET, Erdei N, et al (2008) Tissue-specific regulation of microvascular diameter: opposite functional roles of neuronal and smooth muscle located vanilloid receptor-1. Molecular Pharmacology 73: 1405–1412. 18256211 10.1124/mol.107.043323

[28] Cavanaugh DJ, Chesler AT, Jackson AC, Sigal YM, Yamanaka H, et al (2011) Trpv1 reporter mice reveal highly restricted brain distribution and functional expression in arteriolar smooth muscle cells. J Neurosci 31: 5067–5077. 21451044 10.1523/JNEUROSCI.6451-10.2011 PMC3087977

[29] Czikora Á, Lizanecz E, Bakó P, Rutkai I, Ruzsnavszky F, et al (2012) Structure-activity relationships of vanilloid receptor agonists for arteriolar TRPV1. British Journal of Pharmacology 165: 1801–1812. 21883148 10.1111/j.1476-5381.2011.01645.x PMC3372831

[30] Pinter E, Szolcsanyi J (1995) Plasma extravasation in the skin and pelvic organs evoked by antidromic stimulation of the lumbosacral dorsal roots of the rat. Neuroscience 68: 603–614. 7477970 10.1016/0306-4522(95)00104-q

[31] Szallasi Á, Blumberg PM (1999) Vanilloid (capsaicin) receptors and mechanisms. Pharmacological Reviews 51: 159–212. 10353985

[32] Caterina MJ, Leffler A, Malmberg A, Martin W, Trafton J, et al (2000) Impaired nociception and pain sensation in mice lacking the capsaicin receptor. Science 288: 306–313. 10764638 10.1126/science.288.5464.306

[33] Czikora Á, Lizanecz E, Boczán J, Daragó A, Papp Z, et al (2012) Vascular metabolism of anandamide to arachidonic acid affects myogenic constriction in response to intraluminal pressure elevation. Life Sciences 90: 407–415. 22285599 10.1016/j.lfs.2011.12.016

[34] Khairatkar-Joshi N, Szallasi Á (2009) TRPV1 antagonists: the challenges for therapeutic targeting. Trends in Molecular Medicine 15: 14–22. 19097938 10.1016/j.molmed.2008.11.004

[35] Kissin I, Szallasi A (2011) Therapeutic targeting of TRPV1 by resiniferatoxin, from preclinical studies to clinical trials. Curr Top Med Chem 11: 2159–2170. 21671878 10.2174/156802611796904924

[36] Mezey E, Toth ZE, Cortright DN, Arzubi MK, Krause JE, et al (2000) Distribution of mRNA for vanilloid receptor subtype 1 (VR1), and VR1-like immunoreactivity, in the central nervous system of the rat and human. Proc Natl Acad Sci U S A 97: 3655–3660. 10725386 10.1073/pnas.060496197 PMC16295

[37] Jancso G, Hokfelt T, Lundberg JM, Kiraly E, Halasz N, et al (1981) Immunohistochemical studies on the effect of capsaicin on spinal and medullary peptide and monoamine neurons using antisera to substance P, gastrin/CCK, somatostatin, VIP, enkephalin, neurotensin and 5-hydroxytryptamine. J Neurocytol 10: 963–980. 6171625 10.1007/BF01258524

[38] Nagy JI, Vincent SR, Staines WA, Fibiger HC, Reisine TD, et al (1980) Neurotoxic action of capsaicin on spinal substance P neurons. Brain Res 186: 435–444. 6153557 10.1016/0006-8993(80)90987-7

[39] Helyes Z, Pozsgai G, Börzsei R, Németh J, Bagoly T, et al (2007) Inhibitory effect of PACAP-38 on acute neurogenic and non-neurogenic inflammatory processes in the rat. Peptides 28: 1847–1855. 17698245 10.1016/j.peptides.2007.07.001

[40] Helyes Z, Thán M, Oroszi G, Pintér E, Németh J, et al (2000) Anti-nociceptive effect induced by somatostatin released from sensory nerve terminals and by synthetic somatostatin analogues in the rat. Neuroscience Letters 278: 185–188. 10653024 10.1016/s0304-3940(99)00936-2

[41] Hong S, Wiley JW (2005) Early painful diabetic neuropathy is associated with differential changes in the expression and function of vanilloid receptor 1. The Journal of Biological Chemistry 280: 618–627. 15513920 10.1074/jbc.M408500200

[42] Facer P, Casula MA, Smith GD, Benham CD, Chessell IP, et al (2007) Differential expression of the capsaicin receptor TRPV1 and related novel receptors TRPV3, TRPV4 and TRPM8 in normal human tissues and changes in traumatic and diabetic neuropathy. BMC Neurology 7: 11. 17521436 10.1186/1471-2377-7-11 PMC1892784

